# 5-Arylidene(chromenyl-methylene)-thiazolidinediones: Potential New Agents against Mutant Oncoproteins K-Ras, N-Ras and B-Raf in Colorectal Cancer and Melanoma

**DOI:** 10.3390/medicina55040085

**Published:** 2019-03-31

**Authors:** Cristina Nastasă, Radu Tamaian, Ovidiu Oniga, Brîndușa Tiperciuc

**Affiliations:** 1 Department of Pharmaceutical chemistry, Faculty of Pharmacy, “Iuliu Hațieganu” University of Medicine and Pharmacy, 41 Victor Babeș Street, RO-400012 Cluj-Napoca, Romania; onigao65@yahoo.com (O.O.); brandu32@yahoo.com (B.T.); 2 National Research and Development Institute for Cryogenics and Isotopic Technologies, ICSI Analytics, 4th Uzinei Street, RO-240050 Râmnicu Vâlcea, Romania; 3 SC Biotech Corp SRL, 4th Uzinei Street, RO-240050 Râmnicu Vâlcea, Romania

**Keywords:** thiazolidine-2,4-dione, K-Ras, N-Ras, B-Raf, cytotoxicity, ADME-Tox, molecular docking

## Abstract

*Background and objectives:* Cancer represents the miscommunication between and within the body cells. The mutations of the oncogenes encoding the MAPK pathways play an important role in the development of tumoral diseases. The mutations of KRAS and BRAF oncogenes are involved in colorectal cancer and melanoma, while the NRAS mutations are associated with melanoma. Thiazolidine-2,4-dione is a versatile scaffold in medicinal chemistry and a useful tool in the development of new antitumoral compounds. The aim of our study was to predict the pharmacokinetic/pharmacodynamic properties, the drug-likeness and lead-likeness of two series of synthetic 5-arylidene(chromenyl-methylene)-thiazolidinediones, the molecular docking on the oncoproteins K-Ras, N-Ras and B-Raf, and to investigate the cytotoxicity of the compounds, in order to select the best structural profile for potential anticancer agents. *Materials and Methods:* In our paper we studied the cytotoxicity of two series of thiazolidine-2,4-dione derivatives, their ADME-Tox properties and the molecular docking on a mutant protein of K-Ras, two isoforms of N-Ras and an isoform of B-Raf with 16 mutations. *Results:* The heterocyclic compounds strongly interact with K-Ras and N-Ras right after their posttranslational processing and/or compete with GDP for the nucleotide-binding site of the two GTPases. They are less active against the GDP-bound states of the two targets. All derivatives have a similar binding pattern in the active site of B-Raf. *Conclusions:* The data obtained encourage the further investigation of the 5-arylidene(chromenyl-methylene)-thiazolidinediones as potential new agents against the oncoproteins K-Ras, N-Ras and B-Raf.

## 1. Introduction

Cancers are a group of diseases which can be perceived as miscommunications between the cells and within the cells. One of the most dangerous triggers that leads to cancers is the mutation of genes. Oncogenes are genes that encode proteins able to induce cancer via various metabolic pathways. Somatic mutations in genes encoding the mitogen-activated protein kinase (MAPK) pathways components occur frequently in various tumors, making them critical turning points in the development of human cancer [1]. Oncogenic mutations in MAPK signaling pathways frequently affect the Ras proteins and the serine/threonine-protein kinase B-Raf (B-Raf) in the extracellular signal-regulated kinase pathway [1,2,3]. In this respect, the most common somatic mutations of Kirsten Rat Sarcoma Viral Oncogene Homolog KRAS and BRAF oncogenes are known to play an important role in the advance and progression of both colorectal cancer (CRC) and melanoma [1,2,4,5,6,7,8,9,10,11]. Moreover, Neuroblastoma Rat Sarcoma Viral Oncogene Homolog NRAS mutations also have a crucial role in the development of melanoma [7,9,12] and are becoming an emerging threat in CRC [13]. In a very recent review, Cicenas and collaborators suggested that the mutated oncoproteins of KRAS, NRAS and BRAF oncogenes could skip the normal activation stage [7].

Clinical studies revealed that the prevalence of KRAS mutations in codons 12 and 13 in the tumors of patients with metastatic CRC range from 35% to 42% [4,6,14], meanwhile the three most common mutations (G12D, G12V and G13D) account for approximately 75% of all KRAS mutations [6]. KRAS undergoes alternative splicing, resulting in two isoforms of GTPase KRas (K-Ras) that differ only in the C-terminal region [15,16] and both isoforms (K-Ras4A and K-Ras4B) are oncogenic when gene is mutated [5,15,16,17].

Mutations of NRAS appear in codons 12, 13 and 61 and arise in 15%–20% of all melanomas and the mutant GTPase NRas (N-Ras) has been associated with aggressive clinical behavior and poor prognosis [12].

The products of KRAS and NRAS genes (K-Ras and N-Ras isoforms) belong to the Ras proteins family [7,11,15,18] and are both small GTPases, having a 189 amino acids (AAs) length. Both GTPases are involved in cellular signal transduction, having a crucial role in the regulation of cell proliferation, differentiation and survival through various pathways. K-Ras and N-Ras act as molecular switches by cycling between their guanosine-5′-triphosphate bound (GTP-b) active state and their guanosine 5′-imidotriphosphate bound (GDP-b) inactive state [15,18]. Recently, a 20 AAs length isoform of N-Ras was found to be expressed in an aggressive cell phenotype of melanoma [19]. This 5th isoform of Ras proteins doesn’t have GTPase activity and probably binds to another protein(s), to increase the aggressiveness of melanoma cells.

BRAF gene encodes B-Raf, a protein belonging to the protein kinase superfamily, the tyrosine-kinase like (TKL) serine/threonine-protein kinase family, and the RAF subfamily [7,8]. B-Raf plays an important role in regulating the MAP kinase/ERKs signaling pathway, which affects cell division, differentiation, and secretion. BRAF mutations have been associated with various cancers, somatic missense mutations appearing in 66% of malignant melanomas [8].

The conventional chemotherapy, due to its lack of action selectivity, has many adverse effects. The research conducted in the last years has aimed to achieve a better understanding of the mechanism of genesis and progression of malignant tumors, from where it is outlined the necessity of targeted therapies [20]. Discovering efficient gene inhibitors has become a valuable direction in fighting cancer. Some of the inhibitors may act as covalent binders [21,22,23,24,25,26]. 2,4-Thiazolidinedione (TZD) proved to be a very versatile scaffold in medicinal chemistry; the heterocycle itself or combined with other rings is a highly studied tool in cancer therapy. TZDs act mainly as agonist of the nuclear receptor PPARγ. During recent years, numerous studies have been performed to understand their anticancer mechanism of action. It seems that TZDs exert PPARγ-independent effects on a broad spectrum of signaling targets: Wnt signal transduction pathways, Raf/MEK/ERK and PI3K/Akt signaling pathway, DNAs and RNAs dependent interaction, PIM kinases inhibitor pathways, in producing antiproliferative or apoptopic activity in various cell lines [27,28,29,30,31]. The derivatives substituted with 5-arylidene/5-chromenyl-methylene and 3-benzylidene groups seem to express higher activity [27].

Based on our team’s experience in the virtual screening, molecular docking, chemical synthesis and biological investigation of different heterocyclic-based compounds [32,33,34], we present here the investigation of the cytotoxicity of two series of 5-arylidene (chromenyl-methylene)-thiazolidine-2,4- diones, their ADME-Tox profiling and the molecular docking on K-Ras, N-Ras and B-Raf proteins.

## 2. Materials and Methods

### 2.1. Virtual Screening

VS carried out in this paper used two cheminformatics tools to accomplish its aim: an ADME-Tox predictor and a docking software. 

Ligands: prior to VS, an academic license of MarvinSketch was used for drawing, displaying of 2D structure and 3D optimization of all ligands and generation of the required input files for ADME-Tox predictions (SDF files) and docking (Tripos MOL2 files), MarvinSketch 16.10.24.0, 2016, ChemAxon (https://www.chemaxon.com) [35].

Prediction of the ADME-Tox properties: was done with FAF-Drugs3 [36].

The previously generated SDF files were formatted accordingly FAF-Drugs3’s requirements using the files formatter submodule—Bank Formatter. For the estimation of the lipophilicity and of the derived ADME-Tox descriptors, we used XLOGP3 [37], due to its high prediction precision [38]. A series of FAF-Drugs3′s build-in filters for lead-likeness, drug-likeness, detection of non-peptidic inhibitors of protein-protein interactions (PPIs) [39], detection of undesirable moieties and substructures (UMSs) involved in toxicity problems [40,41,42,43,44,45,46,47,48,49,50], covalent inhibitors [51,52], Pan-Assay Interference Compounds (PAINS) [53,54] and a series of customized filters for safety profiling [44,55,56,57] were exploited for the ADME-Tox screening.

The Lead-Like Soft filter uses descriptors for lead-likeness [58,59,60,61], meanwhile the Drug-Like Soft filter is built on physico-chemical, molecular properties and bioavailability data, commonly encountered in the development of new drugs [58,62,63,64,65]. These soft filters use a build-in statistical analysis of drugs [36] from the e-Drugs3D library [65] for the threshold values of the computed parameters.

PPIs are required in a normal, healthy life, while the abnormal PPIs may lead to diseases. In consequence, they may be considered important targets in medicinal chemistry [66]. For this, FAF-Drugs3 uses a decision tree [39,67] built on two trained Dragon descriptors, Ui and RDF070m [68].

The detection of UMSs involved in toxicity was possible due to FAF-Drugs3 build-in filters, based on the literature data. Also, for identifying the covalent inhibitors (CIs), FAF-Drugs3 build-in filters were applied. Three filters (A, B and C) [36,53,69] were used in order to discover PAINS [45,46].

We investigated the safety profiling according to the GSK 4/400 rule [57], the Pfizer 3/75 rule [56], the estimation of phospholipidosis induction (PhI) [55], the MedChem rules [44] and the golden triangle (GT) rule [70]. The MedChem rules allow the identification of the molecules that may disturb the biological assays, allowing their removal from the screening. We used the MedChem rules in our screening process with the regular settings, involving a 100-demerit cutoff.

### 2.2. Molecular Docking

Targets: docking demands the 3D structure of a target, which contains its spatial coordinates, and for the target identification process it was necessary to cross-reference 3 on-line databases to identify the most adequate targets with a high-resolution 3D structure—a resolution higher than 2.0 Å being advised for the docking computations [71]: The Human Gene Database—GeneCards^®^ (http://www.genecards.org) [72], The Universal Protein Resource—UniProt (http://www.uniprot.org) [73] and RCSB Protein Data Bank—RCSB-PDB (http://www.rcsb.org) [74]. The targets selected were: one of the most common mutants of K-Ras [6], the two isoforms of N-Ras (the canonical form and the short isoform) and an oncogenic B-Raf isoform with 16 mutations (Table 1).

Docking set-up: all ligands were docked against the selected targets (Table 1), in separate runs, with PyRx—Python Prescription 0.9.5 [77] AutoDock Vina was used as the docking algorithm [78]. AutoDock Vina automatically calculates the grid maps [78] based on a scoring function, inspired by an X-score [79] and tuned with PDBbind data set [80,81], to predict noncovalent binding and clusters the results. The renderings of resulting 3D images were performed with the help of The Visualization ToolKit (VTK –Kitware, Inc. USA) (http://www.kitware.com) [82]—an embedded module of PyRx 0.9.5. Supplementary, Molegro Molecular Viewer 2.5 (Molegro, A CLC bio company, Aarhus N, Denmark) was performed for more advanced extraction of data and high resolution renderings of the individual poses. Docking runs against the corresponding PDB IDs (4DSU and 3CON) were carried out twice: with and without the GDP molecule bound in its pocket. Separate single docking runs were carried out for the N-Ras isoform 5 (2N9C) and for B-Raf (5ITA). The docking runs were realized setting an extended search space to completely cover the target, with a volume higher than 27.0 Å3, meanwhile the exhaustiveness was manually expanded 10 times from the default value (8), to increase the precision of all predictions [78,83].

### 2.3. Cytotoxicity

The murine cancer cell lines B16 (mouse melanoma) and CT26 (colorectal carcinoma) (Thermo Fisher Scientific, Waltham, MA, USA) were grown in Dulbecco’s modified essential medium (DMEM). This was supplemented with 10% fetal bovine serum, 2 mM L-glutamine, 100 U/mL penicillin and 100 μg/mL streptomycin. The growing cancer cells were plated onto 96-well plates, at 5000 cells/well, in 200 μL DMEM. After 24 h, the cells were exposed, for 48 h, to dimethylsulfoxide (DMSO) and to the compounds’ solutions, respectively. Stock solutions of compounds (10 mm) were diluted, in order to obtain solutions of different concentrations (100 μm, 50 μm, 25 μm, 12.5 μm, 6.25 μm and 3.125 μm) [34]. The MTT (1-(4,5-dimethylthiazol-2-yl)-3,5-diphenyltetrazolium) test was used to assess the viability of the cells. The number of the living cells, after 72 h of culture, directly proportional to the intensity of the blue color, was spectrophotometrically measured, at 562 nm, by a microplate reader (BioKinetics Reader EL340, Fisher Bioblock Scientific, Illkirch, France) [84]. Control cells were used and they were exposed to 1% DMSO. The experiments were repeated three times. The results obtained were quantified as the inhibitory concentrations for 50% of cells (IC50), for a 48 h exposure time.

## 3. Results

### 3.1. Chemistry

The thiazolidinedione derivatives investigated for their cytotoxicity were previously synthesized, with the exception of compound 26, which was obtained according to the technique [85]:

*5-((6-chloro-4-oxo-4H-chromen-3-yl)methylene)-3-(2-(4-methoxyphenyl)-2-oxoethyl)thiazolidine-2,4-dione* (26)*:* Yield 75%. Yellow powder, mp: 300 °C. ^1^H NMR (DMSO-*d*_6_, 500 MHz,ppm): δ 3.06 (s, 3H, -CH_3_); 5.23 (s, 2H, -CH_2_-); 7.12 (d, 2H, phenyl); 7.65 (d, 1H, C8-chromone-H); 7.71 (dd, 1H, C7-chromone-H); 7.75 (s, 1H, C = CH); 7.94 (s, 1H, C5-Chromone-H); 8.06 (d, 2H, phenyl); 8.95 (s, 1H, C2-chromone-H). Anal. Calcd. (%) for C_22_H_14_ClNO_6_S (455.87): C, 57.96; H, 3.10; N, 3.07; S, 7.03. Found: C, 57.92; H, 3.09; N, 3.06; S, 7.05. MS (EI, 70 eV): m/z: 456.80 [M + 1].

### 3.2. Virtual screening (VS)—ADME-Tox predictions

A potential drug candidate has to correspond to some important drug features, such as: oral absorption, body distribution, metabolism, excretion, low toxicity, beside its pharmacological activity. This is why the virtual screening is extremely important in drug development, allowing the prediction of these parameters (ADME-Tox predictions), with the help of specialized software, before the effective lab synthesis. In our case, a license of MarvinSketch was involved in drawing and generating the 2D structures, 3D optimization of all ligands, and also for creating the input SDF files for the ADMET profiling and Tripos MOL2 files for docking (MarvinSketch 17.6.0, 2017, ChemAxon, Budapest, Hungary) [35]. Table 2 summarizes the results of the ADME-Tox screening carried out with FAF-Drugs3, for the lead-likeness and drug-likeness criteria.

For a drug, a good oral bioavailability is a desired characteristic. The predictors used for this property are: a good intestinal absorption, a reduced molecular flexibility, low polar surface area and the hydrogen-bounding ability.

Thiazolidinediones 1–3 and 7–11 have a higher value of LogP, in terms of the drug-likeness filters (LogP > 5), and only compounds 16–25 respect the lead-like criteria (LogP < 4). Thiazolidinedione derivatives 1–3 and 26–28 also have bad predictions for the molecular weight (MW). All molecules have values of tPSA inferior to 160 Å^2^, passing the criteria requested for the gastro-intestinal absorption, after an oral administration. The studied substances have less than 9 rotatable bonds (RtB) and no chirality center (SC); in consequence, exhibiting low conformational flexibility. All compounds validate all other filters for drug-likeness and also, for lead-likeness (Table 2).

The ADME-Tox profiling also provides a helpful guidance on acute and later toxicity (Table 3).

The risk and safety concern profiling for the studied molecules revealed that these are not phospholipidosis non-inducers. They are free of PAINS. All the lead-like compounds (16–25) were detected as not being PPIs friendly and, with the exception of 16, the rest of all the investigated compounds were flagged as possible covalent inhibitors, due to the presence of the α, β-unsaturated carbonyl [40,86,87].

It can be observed that thiazolidinedione, a high risk UMSs [36], was detected in the structure of 21–25 (Table 3), meanwhile all the other compounds have in their structure other low risk UMSs, like nitro in compounds 12 and 13 [40,42,43], halogenure in 1-3, 7–11, 17, 18, 20, 21, 25–28 and thioester [44]. Moreover, the thioester moiety (present in all compounds) is also considered a liability by the MedChem rules, due to being potentially reactive or promiscuous [44].

The screening revealed that GSK 4/400 rule, the Pfizer 3/75 rule and the GT rule placed the compounds 16, 17, 19, 20, 23 and 24, under the most favorable ADME-Tox predictions.

### 3.3. Molecular Docking

**Figure 1:**
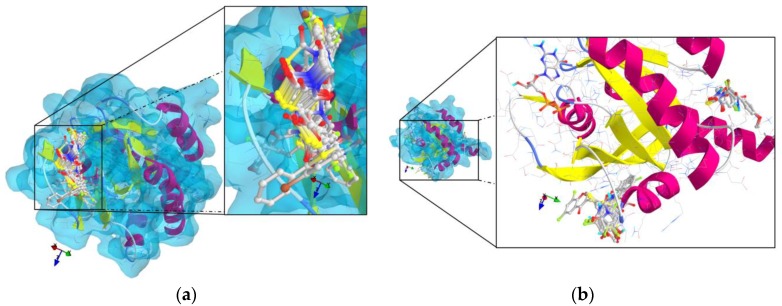
General views and details of the docking poses in the active site of K-Ras (target is presented as thin sticks with secondary structure drawn as cartoon backbone and transparent light blue molecular surface, meanwhile ligands are figured as ball-and-stick—image rendered with VTK/PyRx 0.9.5). (a) The docking results for K-Ras without GDP bound in the pocket; (b): The docking poses from the simulation against the GDP-bound state of K-Ras (top-left: GDP; central-bottom: 1–16, 23, 28; top-right: 17–22, 24–27).

**Figure 2:**
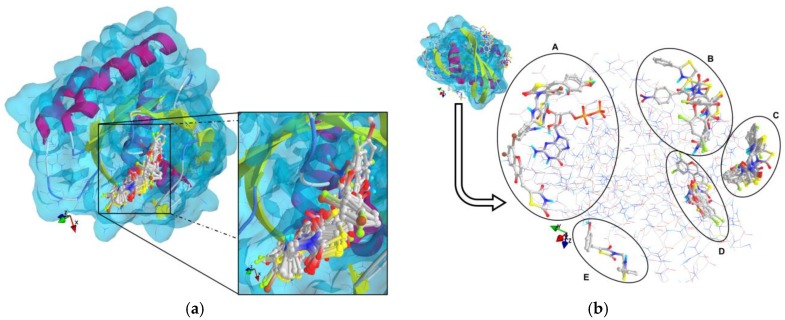
General views and details of the docking poses in the active site of N-Ras (the target is presented as thin sticks with secondary structure drawn as cartoon backbone and transparent light blue molecular surface; the ligands are depicted as ball-and-stick—image rendered with VTK/PyRx 0.9.5). (**a**): The docking results for N-Ras without GDP bound in the pocket; (**b**): The docking poses from the simulation against the GDP-bound state of N-Ras (A: GDP, 3–4; 6, 8, 15, 22; B: 13, 16, 23, 28; C: 1–2, 7, 9–12, 14, 18-20; D: 15, 21, 24–27; E: 5).

**Figure 3:**
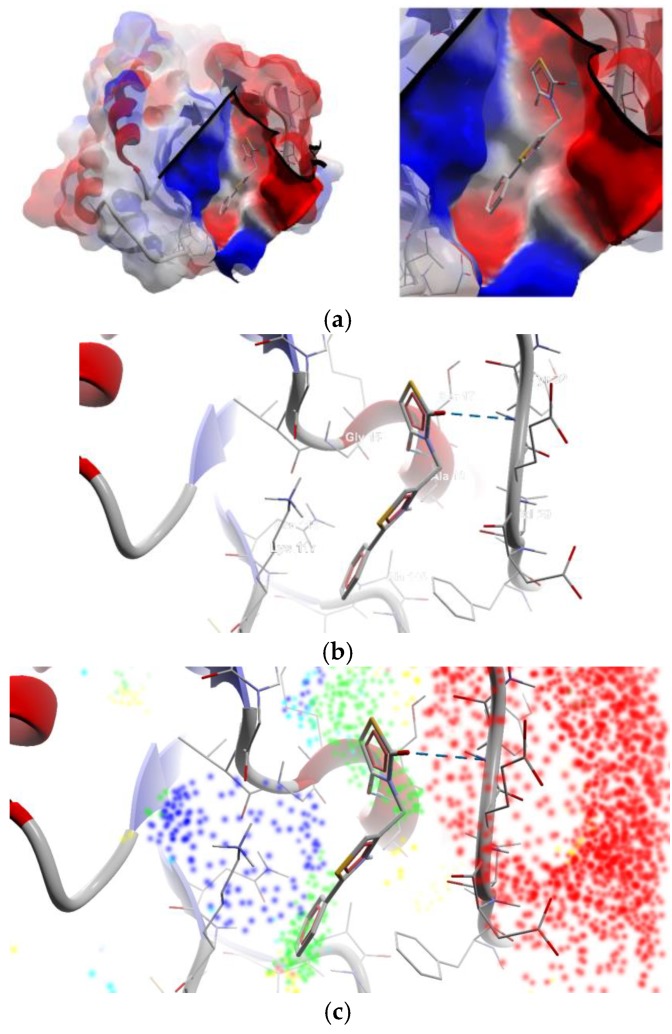
Best pose of compound 16 in the active site of the canonical isoform of N-Ras without GDP bound in the pocket (the target is presented as thin sticks with secondary structure drawn as cartoon backbone; the ligand is drawn as sticks; the H-bonds are in dashed blue lines—image rendered with Molegro Molecular Viewer 2.5). (**a**) General view and detail: ligand is presented buried in the nucleotide-binding pocket (molecular surface of target is depicted as electrostatic potential molecular surface); (**b**) Detail: hydrogen bonds formation with Ala18 and Tyr32; (**c**) Detail: energy grid depiction (green—steric favorable; light blue—hydrogen acceptor favorable; yellow—hydrogen donor favorable; orange to red and dark blue—electrostatic interactions).

**Figure 4:**
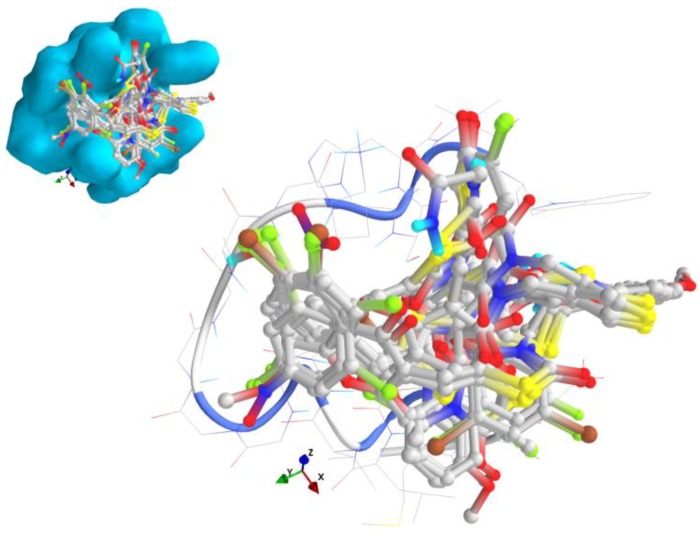
General view and detail of the docking poses in the active site of N-Ras isoform 5 (the target is presented as thin sticks with secondary structure drawn as cartoon backbone and solid light blue molecular surface; the ligands are drawn as ball-and-stick—image rendered with VTK/PyRx 0.9.5).

**Figure 5:**
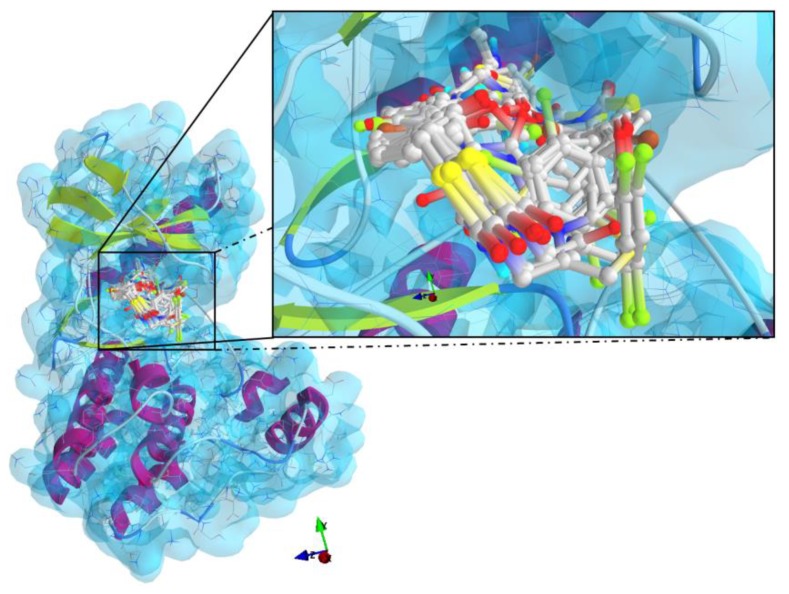
General view and detail of the docking poses in the active site of B-Raf (the target is presented as thin sticks with secondary structure drawn as cartoon backbone and transparent light blue molecular surface; the ligands are drawn as ball-and-stick - image rendered with VTK/PyRx 0.9.5).

The results of the molecular docking runs on the mutant K-Ras, N-Ras or B-Raf isoforms are presented in Table 4 as binding affinity (BA) for the best poses, at root-mean-square deviation (RMSD) equal to zero. The detailed binding patterns and the total energetic interactions are showed in Supplementary Table S1. The graphical depiction of the docking results is illustrated in Figure 1, Figure 2, Figure 3, Figure 4 and Figure 5.

The strongest interaction with the targets chosen was that of compound 12, bearing a nitro moiety: the binding affinity for the mutant isoform of K-Ras was −10 kcal/mol, respectively for N-Ras isoforms, −6.70 kcal/mol and −6.40 kcal/mol. Compound 26 proved to be a strong binder of B-Raf (−10.40 kcal/mol) and compound 27, of N-Ras (−10 kcal/mol). The substances which passed the lead-like filter in the ADME-Tox predictions (16–25) have good binding affinities to the targets, but do not display the strongest interaction. From Table 4 it could be observed that the safer lead-like compound, 16 (the non-covalent binder—Figure 3) is a weak binder of K-Ras and B-Raf, and slightly more potent that 19 against the canonical isoform of N-Ras. All compounds are less active against the N-Ras isoform 5 (which doesn’t have GTPase activity and is responsible for an aggressive phenotype of melanoma) and interact with the alpha-helix (Supplementary Table S1 and Figure 4).

The thiazolidinedione derivatives studied bound more weakly to the GDP-bound state of the GTPases (Figure 1 and Figure 2).

The thiazolidinediones studied have a similar binding pattern in the active site of B-Raf (Figure 5) from the protein kinase domain, between Ile463 and Gly596 (Supplementary Table S1).

### 3.4. Cytotoxicity

Genetic alterations in the MAPK pathway (including the mutation of KRAS, NRAS and BRAF genes) can cooperate in the development of B16 melanomas [88,89] and CT26 colorectal carcinoma [90,91]. Considering this aspect, we investigated the cytotoxicity of the compounds on B16 and CT26 murine cell lines. The results obtained are presented in Table 5.

The best inhibitory effect of the B16 cells was registered for the compound 3 (IC50 = 17.061 μm), bearing a 4-Br-benzylidene fragment in its structure. The weakest activity was that of derivatives 10, 15 and 16, with IC50 > 100 μm. Regarding the effect against the CT26 cells, it can be observed that compound 1, with a 2-Br-benzylidene moiety, had the lowest IC50 (27.227 μm), and therefore had the best inhibitory activity. The weakest effect was displayed by the thiazolidinediones 3, 4, 7, 9–12, 15–19 (IC50 > 100 μm).

## 4. Discussion

The thiazolidinedione derivatives belong to two structural profiles: 5-arylidene-*N*-(phenyl-thiazolyl-methylene)-2,4-thiazolidinediones 1–16 (Table 6) [33] and 5-chromenyl-methylene-2,4-thiazolidinediones 17–28 (Table 7) [34,85,92].

Virtual screening (VS) proved to be a highly useful tool for drug discovery, being able to select the most promising chemical profiles as drug candidates, before the lab synthesis. This adds value to a targeted, more environmental-friendly obtention process, with a considerable reduction of the work time and a remarkable increase in efficiency.

A good oral bioavailability represents a very important characteristic of a bioactive substance. In our case, all the investigated compounds complied with the drug-likeness filters. Even if molecules 1–3 and 7–11 had higher values of LogP, since there was only one violation of Lipinski’s “Rule of 5” (RO5) [93], the estimation of the oral bioavalability was good. Regarding the lead-likeness predictions, only compounds 16–25 can be considered good lead-like molecules, meanwhile all the rest of the investigated structures fail in terms of the logarithm of the partition coefficient between n-octanol and water (LogP). All thiazolidinedione derivatives complied with Veber’s rule [94] and with Egan’s rule [95] on the molecular properties with impact on the oral bioavailability. Topological polar surface area (tPSA), a descriptor that correlates well with the transport through membranes, including the blood-brain barrier, had values inferior to 140 Å^2^, suggesting that the compounds pass the criteria required for a good gastro-intestinal absorption, after an oral administration. Supplementary, all derivatives were predicted to have a reduced blood-brain barrier transport (tPSA > 90 Å^2^. This aspect presents a high significance due to the reduced or totally absent possible side effects on the central nervous system.

In drug development, the investigation of both acute and later toxicity is mandatory: a drug must be pharmacologically active, but also very well tolerated, without side effects. The software-based predictions of the safety and risks profiling are very useful tools in medicinal chemistry, providing many advantages, such as: the availability of the specialized software, the rapidity of the data obtention, the possibility of selecting multiple parameters and filters and the major ethical advantage of reducing the number of the lab animals sacrificed in the classical toxicity assays. The screening performed on our compounds showed that these are not inducers of phospholipidosis, which is a disorder manifested by the accumulation of phospholipids in tissues and a sign of molecules’ toxicity. All substances seemed to be free of PAINS (structures or substructures predicted not to interfere with the biological assays) and PPIs friendly, therefore successfully complying with these safety criteria.

The ADME-Tox profiling identified some low risk problematic moieties in the structure of the compounds, like thiazolidinedione, halogenure, nitro or thioester. MedChem rules considered the thioester fragment as potentially reactive or promiscuous. Considering this observation, the lead-like compounds 16–20 should be placed on the short list of possible hits for further structural optimization in a drug development project. Applying the GSK 4/400 rule, the Pfizer 3/75 rule and the GT rule, we could select the derivatives 16, 17, 19, 20, 23 and 24 as the best candidates, with an optimal permeability (low clearance) and a good metabolic stability [70].

The ADME-Tox data revealed that the studied thiazolidinedione derivatives display good pharmacokinetic properties, but with some limitations. All compounds pass the drug-likeness criteria, while only compounds 16–25 pass the lead-likeness filter. Further structural optimization might be helpful in achieving better ADME-Tox properties.

Molecular docking represents a modern, useful tool in drug discovery. Based on computation methods, it aims to give a prediction of a ligand-receptor complex, therefore suggesting a mechanism of action for the compounds studied.

In our paper, the binding patterns and the binding affinities of the compounds suggest that the substances may strongly interact with K-Ras and N-Ras right after their posttranslational processing and/or compete with GDP for the nucleotide-binding site of the two GTPases. Moreover, all the investigated molecules are less active against the GDP-bound states of the two targets, being weak binders. The compounds interact with the G domain of K-Ras and N-Ras in the bordering region of the nucleotide-binding pocket, in the absence of GDP molecule, acting as a competitor of GDP for its binding pocket. In the presence of GDP in the binding pocket, the compounds not only are weaker binders, but the binding patterns also indicate less pharmacological relevance.

However, AutoDock Vina cannot evaluate the covalent binding capacity of which derivatives are capable (with the exception of **16**), as resulted from the ADME-Tox predictions.

The binding pattern in the active site of B-Raf from the protein kinase domain, similar for all thiazolidinediones investigated, suggests that the tested compounds may also interact with the other members of TKL serine/threonine-protein kinase family, since the interaction region is highly conserved—especially for the RAF subfamily [7,8].

The evaluation of the cytotoxicity is a mandatory step in drug development. For this, we evaluated in vitro the viability of the B16 (mouse melanoma), respectively CT26 (colorectal carcinoma) murine cell lines growth, in an MTT assay. Analyzing the data obtained, we could say that compounds 2, 3, 7, 9, 11, 17, 21 and 22 manifested a good inhibitory effect (IC50 < 50 μm), against the B16 cells. From these, the derivative 3, with a 4-Br-benzylidene fragment in position 5 of the thiazolidinedione ring, displayed the lowest value of IC50. The proliferation of CT26 cells was effectively impeded by compounds 1, 2, 6, 13, 21. The strongest activity was manifested by the thiazolidinedione derivative **1**, bearing the 2-Br-benzylidene fragment bound to the heterocycle. The good inhibitory effect expressed by the two molecules with bromine, confirm the impact of this halogen on the cytotoxic activity, in total agreement with the literature data [27].

## 5. Conclusions

The cytotoxicity assay realized on the two series of 5-arylidene-*N*-(phenyl-thiazolyl-methylene) -2,4-thiazolidinediones and 5-chromenyl-methylene-2,4-thiazolidinediones, respectively, revealed that the compounds expressed good inhibitory effects against the B16 and CT26 cell lines as well. The strongest activity was manifested by the derivatives with a Br-benzylidene fragment in the structure. All the investigated compounds complied with the drug-likeness filters, while substances 16–25 could be considered as lead-like molecules. The thiazolidine-2,4-diones were less active against the GDP-bound states of the K-Ras and N-Ras isoforms chosen as targets, acting as competitors of GDP for its binding pocket. The binding pattern in the active site of B-Raf was common to all the investigated compounds and was highly conserved. All data obtained encourage us to continue work in the field of the thiazolidinedione heterocyclic derivatives with therapeutic properties.

## Figures and Tables

**Table 1:** Targets selected for docking—3D structural data.

Protein	3D Structure Data	
	PBD ID (Mutation)	Resolution * (Å)
K-Ras	4DSU (G12D) [75]	1.70
N-Ras	3CON [TBP]	1.65
	2N9C ^20AAs^ [19]	NA
B-Raf	5ITA (I543A, I544S, I551K, Q562R, L588N, K630S, F667E, Y673S, A688R, L706S, Q709R, S713E, L716E, S720E, P722S, K723G) [76]	1.95

*: resolution is available only for 3D structures determined by X-ray crystallography; the term is not applicable for structures determined by Nuclear Magnetic Resonance (NMR) spectroscopy; ^20AAs^: the 20 AAs length isoform of N-Ras (N-Ras isoform 5), expressed in an aggressive cell phenotype of melanoma; NA: not applicable; TPB: to be published, according to the RCSB-PBD site: http://www.rcsb.org/pdb/explore/explore.do?structureId = 2N9C.

**Table 2:** Virtual screening done with FAF-Drugs3, for lead-likeness and drug-likeness.

ID	MW (Da)	LogP	H_BA_	H_BD_	Tpsa (Å^2^)	RtB	RiB	R_s_	M_x_S	C_s_	HA	H/C	Crg	T_Crg_	SC
DLS_tv_	100–600	−3–6	≤12	≤5	≤180	≤11	≤30	≤6	≤18	3–35	1–15	0.1–1.1	≤3	−2–2	–
LLS_tv_	150–400	−3–4	≤7	≤4	≤160	≤9	≤30	≤4	≤18	3–35	1–15	0.1–1.1	≤3	−2–2	≤2
**1**	457.36	***5.29***	4	0	103.81	4	25	4	6	20	7	0.35	0	0	0
**2**	457.36	***5.29***	4	0	103.81	4	25	4	6	20	7	0.35	0	0	0
**3**	457.36	***5.29***	4	0	103.81	4	25	4	6	20	7	0.35	0	0	0
**4**	394.47	4.25	5	1	124.04	4	25	4	6	20	7	0.35	0	0	0
**5**	394.47	4.25	5	1	124.04	4	25	4	6	20	7	0.35	0	0	0
**6**	394.47	4.25	5	1	124.04	4	25	4	6	20	7	0.35	0	0	0
**7**	412.91	***5.23***	4	0	103.81	4	25	4	6	20	7	0.35	0	0	0
**8**	412.91	***5.23***	4	0	103.81	4	25	4	6	20	7	0.35	0	0	0
**9**	447.36	***5.86***	4	0	103.81	4	25	4	6	20	8	0.40	0	0	0
**10**	447.36	***5.86***	4	0	103.81	4	25	4	6	20	8	0.40	0	0	0
**11**	447.36	***5.86***	4	0	103.81	4	25	4	6	20	8	0.40	0	0	0
**12**	423.46	4.43	7	0	149.63	5	27	4	6	20	9	0.45	0	0	0
**13**	423.46	4.43	7	0	149.63	5	27	4	6	20	9	0.45	0	0	0
**14**	408.49	4.57	5	0	113.04	5	25	4	6	21	7	0.33	0	0	0
**15**	408.49	4.57	5	0	113.04	5	25	4	6	21	7	0.33	0	0	0
**16**	290.36	2.48	4	0	103.81	3	18	3	6	13	6	0.46	0	0	0
**17**	321.74	2.64	5	0	92.89	1	20	2	10	14	7	0.50	0	0	0
**18**	335.76	3.00	5	0	92.89	2	20	2	10	15	7	0.47	0	0	0
**19**	348.31	1.06	7	2	135.98	2	22	2	10	15	9	0.60	0	0	0
**20**	301.32	2.38	5	0	92.89	1	20	2	10	15	6	0.40	0	0	0
**21**	307.71	2.46	5	1	101.68	1	20	2	10	13	7	0.54	0	0	0
**22**	431.06	3.21	5	1	101.68	1	20	2	10	13	8	0.62	0	0	0
**23**	291.25	1.93	5	1	101.68	1	20	2	10	13	7	0.54	0	0	0
**24**	287.29	2.19	5	1	101.68	1	20	2	10	14	6	0.43	0	0	0
**25**	342.15	3.08	5	1	101.68	1	20	2	10	13	8	0.62	0	0	0
**26**	455.87	4.02	7	0	119.19	5	27	3	10	22	9	0.41	0	0	0
**27**	490.31	4.65	7	0	119.19	5	27	3	10	22	10	0.45	0	0	0
**28**	460.29	4.68	6	0	109.96	4	27	3	10	21	9	0.43	0	0	0

DLS_tv_: threshold values of the *Drug-Like Soft* filter; LLS_tv_: threshold values of the *Lead-Like Soft* filter; MW: molecular weight (in Daltons); LogP: the logarithm of the partition coefficient between n-octanol and water; H_BA_: hydrogen bond acceptors; H_BD_: hydrogen bond donors; tPSA: topological Polar Surface Area; RtB: number of rotatable bonds; RiB: number of rigid bonds; R_s_: number of the smallest set of smallest rings; M_x_S: maximum size of the biggest ring system; C_s_: number of carbon atoms; HA: number of heteroatoms; H/C: the ratio between the number of non-carbon atoms and the number of carbon atoms; Crg: number of charged groups; T_Crg_: formal total charge of the compound; SC: stereo centers (– computed only for leads) ***bold & italic values***: violation of RO5, but do not overpass the threshold values of drug-likeness filters; underlined values: overpass the thresholds for lead-likeness filters.

**Table 3:** ADME-Tox profiling—risks and safety concerns.

ID	PPIs	UMSs	CIs	PAINS Filters			GSK 4/400 Rule	Pfizer 3/75 Rule	PhI	Med Chem	GT Rule
				A	B	C					
**1**	yes	halogenure thioester	UC	ND	ND	ND	bad	warning	not	thioester	out
**2**	yes	halogenure thioester	UC	ND	ND	ND	bad	warning	not	thioester	out
**3**	yes	halogenure thioester	UC	ND	ND	ND	bad	warning	not	thioester	out
**4**	yes	thioester	UC	ND	ND	ND	good	warning	not	thioester	out
**5**	yes	thioester	UC	ND	ND	ND	good	warning	not	thioester	out
**6**	yes	thioester	UC	ND	ND	ND	good	warning	not	thioester	out
**7**	yes	halogenure thioester	UC	ND	ND	ND	bad	warning	not	thioester	out
**8**	yes	halogenure thioester	UC	ND	ND	ND	bad	warning	not	thioester	out
**9**	yes	halogenure thioester	UC	ND	ND	ND	bad	warning	not	thioester	out
**10**	yes	halogenure thioester	UC	ND	ND	ND	bad	warning	not	thioester	out
**11**	yes	halogenure thioester	UC	ND	ND	ND	bad	warning	not	thioester	out
**12**	yes	nitro thioester	UC	ND	ND	ND	bad	warning	not	thioester	out
**13**	yes	nitro thioester	UC	ND	ND	ND	bad	warning	not	thioester	out
**14**	yes	thioester	UC	ND	ND	ND	bad	warning	not	thioester	out
**15**	yes	thioester	UC	ND	ND	ND	bad	warning	not	thioester	out
**16**	no	thioester	ND	ND	ND	ND	good	good	not	thioester	in
**17**	no	halogenure thioester	UC	ND	ND	ND	good	good	not	thioester	in
**18**	no	halogenure thioester	UC	ND	ND	ND	good	warning	not	thioester	in
**19**	no	thioester	UC	ND	ND	ND	good	good	not	thioester	in
**20**	no	thioester	UC	ND	ND	ND	good	good	not	thioester	in
**21**	no	halogenure thioester thiazolidinedione	UC	ND	ND	ND	good	good	not	thioester	out
**22**	no	halogenure thioester thiazolidinedione	UC	ND	ND	ND	good	warning	not	thioester	out
**23**	no	thioester thiazolidinedione	UC	ND	ND	ND	good	good	not	thioester	in
**24**	no	thioester thiazolidinedione	UC	ND	ND	ND	good	good	not	thioester	in
**25**	no	halogenure thioester thiazolidinedione	UC	ND	ND	ND	good	warning	not	thioester	in
**26**	yes	halogenure thioester	UC	ND	ND	ND	bad	warning	not	thioester	out
**27**	yes	halogenure thioester	UC	ND	ND	ND	bad	warning	not	thioester	out
**28**	yes	halogenure thioester	UC	ND	ND	ND	bad	warning	not	thioester	out

PPIs: protein-protein interactions; underlined values: high risk UMSs; Cis: covalent inhibitors; UC: α, β-unsaturated carbonyl (a covalent inhibitor moiety); PAINS: Pan-Assay Interference Compounds; ND: none detected (compound is free of problematic substructures for the corresponding risk criteria); PhI: phospholipidosis induction.

**Table 4:** The binding affinity (BA) expressed by the thiazolidinedione derivatives **1**–**28**.

Ligand ID	BA (kcal/mol)					
	*K-Ras*		*N-Ras*			*B-Raf*
	4DSU	4DSU^GDP−b^	3CON	3CON^GDP−b^	2N9C	5ITA
**1**	−8.00	−7.80	−8.70	−6.10	−5.80	−9.10
**2**	−9.00	−8.00	−8.50	−6.20	−6.30	−9.70
**3**	−8.10	−7.60	−8.70	−5.90	−6.20	−8.90
**4**	−8.00	−7.70	−8.40	−6.50	−5.70	−9.10
**5**	−8.70	−7.70	−9.50	−6.30	−5.80	−9.40
**6**	−9.40	−7.80	−8.40	−6.30	−6.10	−9.10
**7**	−8.40	−7.90	−8.50	−6.40	−6.20	−9.30
**8**	−8.60	−7.80	−8.90	−6.20	−6.30	−9.10
**9**	−8.10	**−8.20**	−8.70	−6.50	−6.20	−9.40
**10**	−8.00	−7.70	−8.70	−6.00	−6.20	−9.40
**11**	−8.30	−7.40	−8.70	−6.20	−5.90	−9.20
**12**	**−10.00**	−7.90	−9.50	**−6.70**	**−6.40**	−9.70
**13**	−8.50	−7.40	−9.30	−6.20	−5.90	−9.80
**14**	−8.40	−7.80	−8.50	−6.20	−5.80	−9.20
**15**	−8.70	−7.60	−8.60	−6.20	−5.80	−9.10
**16**	−8.00	−6.20	−7.80	−5.50	−4.80	−7.90
**17**	−8.50	−6.70	−7.80	−5.80	−5.20	−8.20
**18**	−7.90	−6.60	−7.50	−5.80	−5.10	−8.10
**19**	−8.70	−6.90	−8.70	−6.10	−5.00	−8.20
**20**	−8.70	−6.90	−8.00	−6.10	−5.20	−8.90
**21**	−8.30	−6.80	−8.20	−6.00	−5.20	−9.00
**22**	−8.60	−6.90	−8.20	−5.60	−5.00	−8.60
**23**	−8.60	−7.20	−8.40	−6.30	−5.30	−9.50
**24**	−8.60	−7.00	−8.50	−6.20	−5.30	−9.30
**25**	−8.90	−6.80	−8.50	−5.90	−5.10	−9.00
**26**	−8.70	−7.50	−8.90	−6.60	−5.80	**−10.40**
**27**	−9.10	−7.50	**−10.00**	−6.60	−5.90	−10.20
**28**	−8.80	−7.40	−8.60	−6.40	−6.20	−10.30

**^GDP^*^−^*^b^**: GDP-bound state of GTPases; **bold** values: the strongest interaction with the target chosen.

**Table 5:** The cytotoxicity of the thiazolidinedione derivatives.

Compound	IC50 (μM)	
	B16	CT26
**1**	82.794	***27.227***
**2**	**45.394**	**29.923**
**3**	***17.061***	>100
**4**	74.131	>100
**5**	53.407	57.544
**6**	52.481	**29.444**
**7**	**30.2**	>100
**8**	69.502	81.846
**9**	**37.931**	>100
**10**	>100	>100
**11**	**49.888**	>100
**12**	87.297	>100
**13**	66.834	**40.832**
**14**	62.373	67.298
**15**	>100	>100
**16**	>100	>100
**17**	**28.314** [34]	>100 [34]
**18**	66.374 [34]	>100 [34]
**19**	56.624	>100
**20**	69.823 [34]	62.951 [34]
**21**	**40.272** [34]	**33.651** [34]
**22**	**42.17** [34]	52.481 [34]
**23**	72.277 [34]	73.451 [34]
**24**	85.114 [34]	67.608 [34]
**25**	NT	NT
**26**	NT	NT
**27**	NT	NT
**28**	NT	NT

Bold values: good inhibitory activity; *bold & italic values*: the best inhibitory activity; NT: not tested.

**Table 6:**
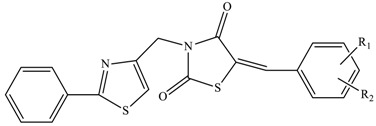
Chemical structures of the *N*-substituted 5-arylidene-2,4-thiazolidinediones 1–16.

Compound	R_1_	R_2_
**1**	2-Br	H
**2**	3-Br	H
**3**	4-Br	H
**4**	2-OH	H
**5**	3-OH	H
**6**	4-OH	H
**7**	3-Cl	H
**8**	4-Cl	H
**9**	2-Cl	3-Cl
**10**	2-Cl	4-Cl
**11**	2-Cl	6-Cl
**12**	3-NO_2_	H
**13**	4-NO_2_	H
**14**	3-OCH_3_	H
**15**	4-OCH_3_	H
**16**	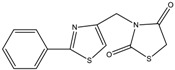	

**Table 7:**
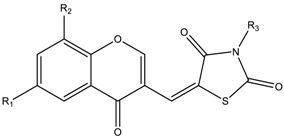
Chemical structures of the 5-chromenyl-methylene-2,4-thiazolidinediones **17**–**28**.

Compound	R_1_	R_2_	R_3_
**17**	Cl	H	CH_3_
**18**	Cl	H	C_2_H_5_
**19**	F	H	CH_2_-CO-NH_2_
**20**	CH_3_	H	CH_3_
**21**	Cl	H	H
**22**	Br	Br	H
**23**	F	H	H
**24**	CH_3_	H	H
**25**	Cl	Cl	H
**26**	Cl	H	CH_2_-CO-C_6_H_4_-OCH_3_ (p)
**27**	Cl	Cl	CH_2_-CO-C_6_H_4_-OCH_3_ (p)
**28**	Cl	Cl	CH_2_-CO-C_6_H_5_

## Supplementary Materials

The following are available online at https://www.mdpi.com/1010-660X/55/4/85/s1, Table S1: Binding patterns and total energetic interactions of the thiazolidine-2,4-dione derivatives.
